# Cell dehydration of intergeneric hybrid induces subgenome‐related specific responses

**DOI:** 10.1111/ppl.13855

**Published:** 2023-01-31

**Authors:** Tomasz Hura, Katarzyna Hura, Kinga Dziurka, Agnieszka Ostrowska, Karolina Urban

**Affiliations:** ^1^ Polish Academy of Sciences The Franciszek Górski Institute of Plant Physiology Kraków Poland; ^2^ Department of Plant Breeding, Physiology and Seed Science, Faculty of Agriculture and Economics Agricultural University Kraków Poland

## Abstract

The aim was to identify subgenome‐related specific responses in two types of triticale, that is, of the wheat‐dominated genome (WDG) and rye‐dominated genome (RDG), to water stress induced in the early phase (tillering) of plant growth. Higher activity of the primary metabolism of carbohydrates is a feature of the WDG type, while the dominance of the rye genome is associated with a higher activity of the secondary metabolism of phenolic compounds in the RDG type. The study analyzed carbohydrates and key enzymes of their synthesis, free phenolic compounds and carbohydrate‐related components of the cell wall, monolignols, and shikimic acid (ShA), which is a key link between the primary and secondary metabolism of phenolic compounds. Under water stress, dominance of the wheat genome in the WDG type was manifested by an increased accumulation of the large subunit of Rubisco and sucrose phosphate synthase and a higher content of raffinose and stachyose compared with the RDG type. In dehydrated RDG plants, higher activity of *L*‐phenylalanine ammonia lyase (PAL) and *L*‐tyrosine ammonia lyase (TAL), as well as a higher level of ShA, free and cell wall‐bound *p*‐hydroxybenzoic acid, free homovanillic acid, free sinapic acid, and cell wall‐bound syringic acid can be considered biochemical indicators of the dominance of the rye genome.

## 1 INTRODUCTION

Together with the progress of climate change, grows the importance of intergeneric hybrids in breeding projects aimed at obtaining new cultivars or crop species with increased tolerance to environmental stresses, including soil drought (Ali et al., 2020; Rauf et al., 2016; Waqar et al., 2014).

Triticale (× *Triticosecale* Witt.), a cross of common wheat (*Triticum aestivum* L.) and rye (*Secale cereale* L.) is a classic example of an intergeneric hybrid (Niedziela et al., 2016). This artificially created cereal was supposed to combine the high productivity of wheat and resistance to biotic and abiotic stresses of rye. This objective was achieved by obtaining secondary forms of hexaploid triticale (AABBRR), with wheat (AABB) and rye (RR) genomes (Hura et al., 2009a; Mergoum et al., 2019; Niedziela et al., 2014). Since then, hexaploid triticale has become an interesting model species to investigate the biology of hybrids under environmental stress (Hura et al., 2009b; Szechyńska‐Hebda et al., 2015; Wąsek et al., 2022; Żur et al., 2013). The investigations include physiological, biochemical, and molecular responses to soil drought and the specific roles the wheat and rye genomes play in these responses (Hura, Dziurka, et al., 2017; Ostrowska et al., 2019). Water stress also identified specific plant properties that result from the interaction of both wheat (AABB) and rye (RR) genomes and that can be uniquely attributed to the triticale genome (AABBRR; Hura et al., 2022).

Earlier studies have shown that the wheat‐dominated genome (WDG) in triticale adaptation to water stress in the generative phase is responsible for; for example, higher activity of the photosynthetic apparatus, accumulation of proteins related to photosynthetic fixation of CO_2_ or higher level of sugars than in the genotypes with the rye‐dominated genome (RDG). Typical responses of the latter to water stress are an increase in the content of cell wall‐bound phenolics or the activity of *L*‐phenylalanine ammonia lyase (PAL) and *L*‐tyrosine ammonia lyase (TAL; Hura et al., 2016; 2022; Ostrowska et al., 2019). Other adaptive responses of triticale to water stress, visible to the naked eye and indicating the dominance of either genome, maybe the manner of flag leaf rolling or aphid colonization of the flag leaves associated with the content of phenolic compounds in the cell wall structures (Hura et al., 2022). The activity of the triticale genome under water stress, manifested by increased content of cell wall‐bound phenolics, was confirmed by identification of loci related to this process on 3R and 6R rye chromosomes. These loci harbor *serine/threonine kinase* and *cytokinin oxidase/dehydrogenase 3* genes that may be involved in the complex process of incorporation of phenolic compounds in the cell wall of triticale leaves (Hura, Tyrka, et al., 2017).

So far, the activity of individual triticale genomes under water stress was mainly analyzed during the phase of generative growth, and clear and specific responses of WDG and RDG type due to the activity of either the wheat or the rye genome were confirmed. In addition, specific features and responses resulting from the interplay between these two genomes were identified (Hura et al., 2022).

Therefore, this study aims to analyze specific responses of the wheat and rye genomes during leaf dehydration in the early growth phase of triticale. Such an approach should allow us to select the appropriate growth phase of the intergeneric hybrid for further detailed research on the activity of individual genomes and the interactions between them. We analyzed the primary metabolism related to the synthesis of carbohydrates, the secondary metabolism related to the synthesis of phenolic compounds, and the content of shikimic acid (ShA), which is a key link between the primary and secondary metabolism of phenolic compounds. The intensity of water stress was analyzed by measuring the water potential in leaf cells, the level of abscisic acid (ABA), and stomatal conductance.

## 2 MATERIALS AND METHODS

### 2.1 Plant material

The study involved two lines of triticale doubled haploid (DH), Hewo (Plant Breeding Strzelce Ltd., Co.) and Magnat (DANKO Plant Breeders Ltd.). Our previous studies showed that under water stress, the DH line Hewo has a WDG type, and the DH Magnat line has a RDG type (Hura et al., 2022; Hura, Tyrka, et al., 2017).

### 2.2 Plant growth conditions

The seeds of both triticale types, WDG and RDG, were sown into 3.7 L pots filled with a mixture of soil and sand (1:3; vol/vol). Following emergence (one leaf stage), the plants were vernalized for 8 weeks at 4°C, photosynthetic photon flux density (PPFD) 150 μmol m^−2^ s^−1^, photoperiod 10‐h light/14‐h darkness. Then the seedlings at the two‐leaf stage were transferred into a greenhouse chamber. Air temperature in the chamber was 25–30°C/15–20°C day/night, and relative air humidity was about 30%. The plants were additionally illuminated (high‐pressure sodium lamps, 400 W; Philips SON‐T AGRO) and the PPFD was about 200–250 μmol m^−2^ s^−1^ (QSPAR Quantum Sensor, Hansatech Instruments LTD). The plants were irrigated with Hoagland's nutrient solution once per week (Hoagland, 1948).

### 2.3 Soil drought conditions

Soil drought was applied to both triticale types at the stage of four leaves (beginning of tillering). By withholding watering, the water content in the pots was gradually reduced to about 30% for 7 days and kept at this level for the next 14 days (in total 21 days of limited watering). Water content in the control pots was maintained at about 75%. Soil moisture in the pots was inspected daily, between 8.00 a.m. and 10.00 a.m., using a gravimetric method and taking into account plant weight (soil water content in the pots was immediately restored to 30%—drought or 75%—control).

### 2.4 Measurements and preparation of plant samples

The measurements were performed on Day 21 of the soil drought. For the analysis, we collected a fully expanded leaf, that is, the fifth one from the bottom. For biochemical and molecular measurements, leaf samples were collected and immediately frozen in liquid nitrogen, lyophilized, and then powdered using stainless steel beads (MM400, Retsch).

#### 2.4.1 Leaf water potential (Ψ_w_)

The leaf water potential was analyzed with an HR‐33 T dew point microvoltmeter (Wescor, Inc.). Leaf discs (5 mm in diameter) were cut, placed inside the thermocouple psychrometer chamber (C‐52) and allowed to reach temperature and water vapor equilibrium for 60 min before measurements were made by the dew point method.

#### 2.4.2 Stomatal conductance (g*_s_)*

Stomatal conductance was recorded using an SC‐1 leaf porometer (Decagon Devices Inc.) on a fully expanded mature leaf.

#### 2.4.3 ABA content

ABA content was assessed according to the procedure described by Hura, Dziurka, et al. (2017). Ultrahigh‐performance liquid chromatography (UHPLC) and an Agilent Infinity 1260 system coupled with 6410 Triple Quad liquid chromatography‐mass spectrometry (MS) with an electrospray interface (ESI; Agilent Technologies) were used.

#### 2.4.4 Protein accumulation

We investigated the proteins responsible for photosynthetic fixation of CO_2_ (RbcL—Rubisco large subunit, forms I and II; Agrisera AS03 037) and sucrose phosphate synthase (SPS; global; Agrisera AS03 035A), a protein associated with carbohydrate metabolism. Protein extraction, electrophoresis, western blot, and gel analysis were carried out as described in our previous paper (Hura et al., 2022).

#### 2.4.5 Carbohydrates

The content of free sugars and starch was analyzed according to Hura et al. (2016). Fructooligosaccharides were estimated following the protocol by Mikuła et al. (2021). HPLC analyses of free sugars, starch, and fructooligosaccharide hydrolysates were done using the Agilent 1200 system (Agilent Technologies) coupled with an ESA Coulochem II 5200A electrochemical detector HPLC (ESA). Separating sugars and starch hydrolysates were done using an RCX‐10; 7 μm; 250 × 4.1 mm column (Hamilton, Ohio) in gradient mode of 75 mM NaOH solution, and 500 mM sodium acetate in 75 mM NaOH solution (Hura et al., 2016).

The average degree of polymerization (DP_av_) of the fructans/oligofructans was calculated using the following formula: DP_av_ = 1 + (F_f_/G_f_), where F_f_ and G_f_ are molar concentrations of fructose and glucose released after enzymatic treatment of fructans/oligofructans (Verspreet et al., 2012).

#### 2.4.6 Shikimic acid

ShA was estimated in the same extract as monolignols, excluding SPE sample cleanup. After solvent exchange to 2% MeOH, a clear centrifuged solution was evaporated and resuspended in 100 μL acetonitrile (ACN). The content of ShA was measured utilizing UHPLC‐tandem MS (MS/MS) in negative ion mode MRM (multiple reaction monitoring). Chromatographic separation was done on Poroshell 120 HILIC‐Z 2.1 × 100 mm, 2.7 μm column (Agilent Technologies), in gradient mode (A–ACN with 5% 20 mM HCOONH_4_; B–20 mM HCOONH_4_ in H_2_O) at 0.5 mL min^‐1^ and 40°C. [2H5]benzoic acid (D‐BeA) was used as ISTD (isotope‐labeled internal standard solution). Further technical details are given in Table S1.

#### 2.4.7 PAL and TAL activity, phenolics content

PAL and TAL activity was measured according to the methods described by Hura et al. (2022). The enzyme activity was expressed as nmol of cinnamic acid (PAL) or *p*‐coumaric acid produced during 1 h per 1 mg of protein. Protein content was determined by the method of Bradford (1976).

The content of free and cell wall‐bound phenolics was estimated according to Hura et al. (2016). Phenolics content was analyzed on an Agilent Infinity 1260 UHPLC column with a fluorescence detector. Specifically, the phenolics were separated on a Zorbax Eclipse Plus Phenyl‐Hexyl 3.5 μm 3.0 × 100 mm column (Agilent Technologies) under a linear gradient of 2% (vol/vol) aqueous solution of formic acid versus MeOH. To maximize sensitivity, the excitation and emission wavelengths were dynamically changed. Details are given in Gołębiowska‐Pikania et al. (2019).

#### 2.4.8 Monolignols

The samples were extracted as described for phytohormones (Hura, Dziurka et al., 2017) and cleaned up in the same manner as for aromatic acids (Hura et al., 2016). D‐BeA was added as an internal standard prior to extraction. The compounds of interest (*p*‐coumaryl alcohol, coniferyl alcohol, and sinapyl alcohol) were measured employing UHPLC‐MS/MS (Agilent Technologies) in positive ion mode MRM. An Ascentis Express RP‐Amide analytical column (2.7 μm, 2.1 × 150 mm; Supelco, Bellefonte) in a linear gradient of 3% ACN in H_2_O versus ACN with 0.01% (vol/vol) HCOOH was used. Further technical details are given in Table S1.

### 2.5 Statistical analysis

Statistical analysis was carried out using Statistica v. 13.0 (Statsoft Inc.). A two‐way analysis of variance (ANOVA) was performed to evaluate the effect of drought and cultivar on each tested parameter. Duncan's multiple range test at the 0.05 probability level was chosen to determine the significance of differences among treatment means.

## 3 RESULTS

In Table 1, we present changes in water potential (Ψ_w_), stomatal conductivity (*g_s_*) and the content of ABA in WDG and RDG triticale exposed to water stress (WS). At a similar level of cell dehydration, ABA content was significantly higher in the WDG type (3827.5 pg mg^−1^ DW) than in the RDG type (2188.1 pg mg^−1^ DW). Under optimal growth conditions, both types showed a similar cell hydration level and ABA content. Stomatal conductivity (*g*_s_) under water stress conditions was approximately two times lower in WDG than in RDG plants (Table 1).

**TABLE 1:** Changes in water potential (Ψ_w_; MPa), content of abscisic acid (ABA; pg mg^−1^ [DW]) and stomatal conductance (*g*_s_; mmol (H_2_O)/m^2^/s) in triticale with wheat‐dominated genome (WDG) and rye‐dominated genome (RDG).

Parameters	Treatment	WDG	RDG
Ψ_w_	C	−0.85 ± 0.03a	−0.82 ± 0.04a
	WS	−1.89 ± 0.08b	−1.88 ± 0.06b
ABA	C	444.2 ± 10.9a	378.5 ± 18.3a
	WS	3827.5 ± 120.7b	2188.1 ± 19.9c
*g*_s_	C	361.1 ± 17.2a	350.8 ± 18.3a
	WS	35.9 ± 1.8b	69.0 ± 3.1c

*Note*: Mean values ± SE (*n* = 7). Means indicated with the same letters are not significantly different within measurements (Duncan's multiple range test at *p* = 0.05).

Abbreviations: C, control; WS, water stress.

**FIGURE 1:**
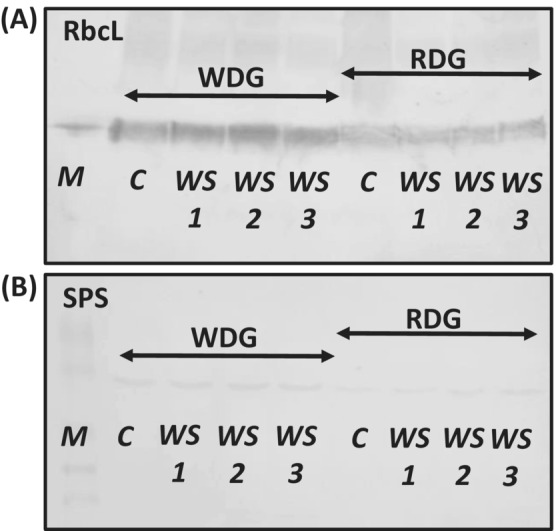
Western blot analyses showing accumulation of larger subunit of Rubisco (RbcL) (A) and sucrose phosphate synthase (SPS) (B) in triticale with wheat‐dominated genome (WDG) and rye‐dominated genome (RDG). M, weight marker; C, control; WS, water stress; 1, 2, 3—samples.

Western blot analysis indicated higher levels of large Rubisco subunit (RbcL; Figure 1A) and SPS (Figure 1B) in WDG versus RDG type. However, water stress (WS) did not evoke any notable changes in the accumulation of these proteins in either triticale type as compared with the control (C).

**FIGURE 2:**
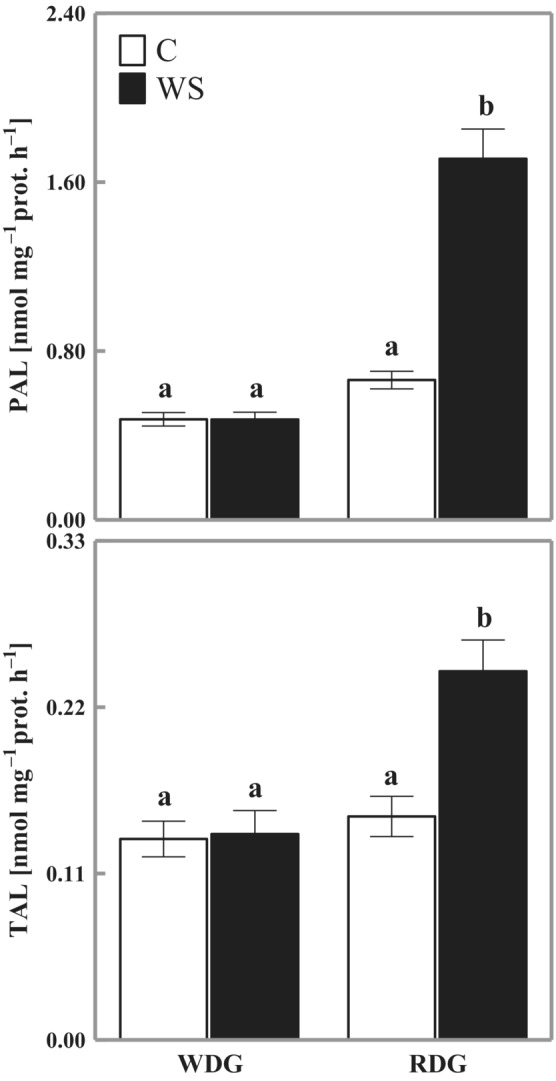
Changes in the activity of *L*‐phenylalanine ammonia lyase (PAL) and *L*‐tyrosine ammonia lyase (TAL) in triticale with wheat‐dominated genome (WDG) and rye‐dominated genome (RDG). C, control; WS, water stress. Mean values ± SE (*n* = 7). Means indicated with the same letters are not significantly different (Duncan's multiple range test at *p* = 0.05).

A significant increase in the activity of PAL (259% of control) and TAL (165% of control) under water stress was only observed for RDG triticale. Water stress did not affect PAL or TAL activity in the WDG type (Figure 2).

**FIGURE 3:**
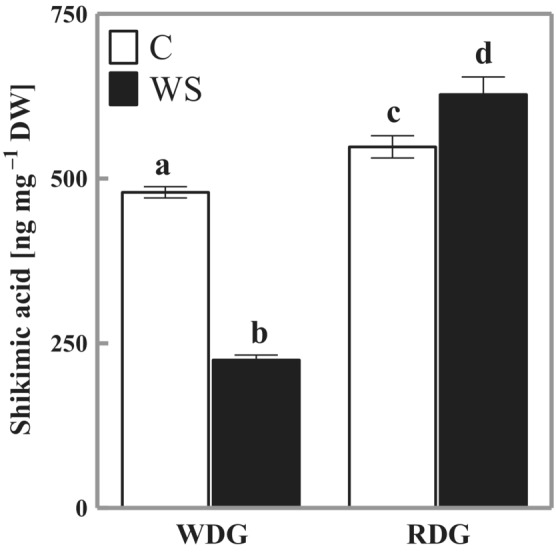
Changes in the content of shikimic acid in triticale with wheat‐dominated genome (WDG) and rye‐dominated genome (RDG). C, control; WS, water stress. Mean values ± SE (*n* = 5). Means indicated with the same letters are not significantly different (Duncan's multiple range test at *p* = 0.05).

Figure 3 presents changes in the content of ShA in WDG and RDG plants. It was significantly higher in RDG triticale both under optimal soil water content (C) and water stress (WS). Interestingly, water stress evoked contrary responses in the investigated intergeneric hybrids, a significant drop in ShA content in WDG plants (47% of control) and a significant rise in RDG ones (115% of control).

Quantitative analyzes of soluble carbohydrates revealed similar and specific changes in their levels (Table 2). Under water stress (WS) conditions, enhanced accumulation of such sugars as glucose, fructose, and stachyose and reduction in sucrose and raffinose were detected in both WDG and RDG plants. Water stress‐induced specific changes in sugar levels were noted for maltose, 111‐5‐kestose, and nystose and included their increased content in WDG type and decreased content in RDG plants. Additionally, in dehydrated leaves of WDG plants, we found a lowered level of kestose (52% of control), but no changes for this carbohydrate were determined for RDG plants. At the same time, at optimal soil water content (C), the WDG type showed a higher content of seven sugars (glucose, fructose, sucrose, maltose, raffinose, 111‐5‐kestose, and nystose) than the RDG type. Under water stress, seven soluble sugars (glucose, fructose, sucrose, maltose, kestose, 111‐5‐kestose, and nystose) were significantly lower in WDG than in RDG triticale (Table 2).

**TABLE 2:** Changes in the content of soluble carbohydrates (nmol/mg [DW]) in triticale with wheat‐dominated genome (WDG) and rye‐dominated genome (RDG).

	Soluble carbohydrates	
	WDG	RDG
Glucose		
C	31.3 ± 2.28a	22.8 ± 0.70c
WS	39.4 ± 0.74b	59.6 ± 0.62d
Fructose		
C	14.2 ± 0.89a	12.2 ± 0.46a
WS	22.6 ± 1.22b	41.4 ± 0.64c
Sucrose		
C	85.3 ± 0.55a	67.6 ± 1.10c
WS	42.3 ± 0.56b	44.6 ± 0.18d
Maltose		
C	7.2 ± 0.18a	2.4 ± 0.06c
WS	4.3 ± 0.09b	7.3 ± 0.02a
Raffinose		
C	2.06 ± 0.037a	1.56 ± 0.038c
WS	1.05 ± 0.014b	0.59 ± 0.020d
Stachyose		
C	0.39 ± 0.038a	0.37 ± 0.019a
WS	0.71 ± 0.016b	0.63 ± 0.005c
Kestose		
C	4.59 ± 0.049a	5.01 ± 0.080c
WS	2.40 ± 0.048b	4.96 ± 0.111c
111‐5‐kestose		
C	1.16 ± 0.06a	0.31 ± 0.01b
WS	0.41 ± 0.04b	0.62 ± 0.05c
Nytose		
C	4.32 ± 0.36a	0.36 ± 0.15b
WS	0.67 ± 0.23b	1.62 ± 0.23c

*Note*: Mean values ± SE (*n* = 5). Means indicated with the same letters are not significantly different for individual carbohydrates (Duncan's multiple range test at *p* = 0.05).

Abbreviations: C, control; WS, water stress.

Our analysis revealed a significant rise in the total amount of fructans in water stress‐exposed RDG plants and a significant drop in WDG plants (Table 3). In the RDG type, we detected a significant increase in the average degree of fructan polymerization from 1.51 (C) to 1.59 (WS), while in the WDG type, a reverse pattern was seen, that is, a decrease from 1.80 (C) to 1.56 (WS) (Table 3). In both WDG and RDG plants, we noted a significant increase in the content of oligofructans induced by water stress, accompanied by a significant decrease in the average DP for WDG (from 1.70 to 1.42), and a significant increase in this parameter for RDG type (from 1.24 to 1.44). Moreover, in both types of triticale, a significant reduction in starch content occurred in dehydrated leaves (5.6% of control in WDG, 12.5% of control in RDG) (Table 3).

**TABLE 3:** Changes in total amount of fructans (nmol/mg [DW]), oligofructans (nmol/mg [DW]), total fructan average degree of polymerization (DP_av_), oligofructan average degree of polymerization (DP_av_), and starch content (μg/mg [DW]) in triticale with wheat‐dominated genome (WDG) and rye‐dominated genome (RDG).

Parameters	WDG	RDG
Total amount of fructans		
C	251.3 ± 4.3a	233.3 ± 3.7a
WS	209.5 ± 9.4b	275.9 ± 5.9c
Amount of oligofructans		
C	131.5 ± 4.9a	147.8 ± 3.1b
WS	149.9 ± 5.1b	182.7 ± 6.4c
Total fructans average degree of polymerization		
C	1.80 ± 0.017a	1.51 ± 1.008c
WS	1.56 ± 0.017b	1.59 ± 0.019b
Oligofructans average degree of polymerization		
C	1.70 ± 0.031a	1.24 ± 0.013c
WS	1.43 ± 0.027b	1.44 ± 0.027b
Starch		
C	1.96 ± 0.083a	2.00 ± 0.017a
WS	0.11 ± 0.008b	0.25 ± 0.010c

Abbreviations: C, control; WS, water stress.

*Note*: Mean values ± SE (*n* = 5). Means indicated with the same letters are not significantly different for individual carbohydrates (Duncan's multiple range test at *p* = 0.05).

Table 4 presents the content of individual phenolic compounds, both soluble and bound with cell wall carbohydrates. In WDG and RDG plants, water stress (WS) induced a significant accumulation of six phenolic compounds: *p*‐hydroxybenzoic acid, vanillic acid, homovanillic acid, ferulic acid, sinapic acid, and rosmarinic acid. In the case of chlorogenic acid and salicylic acid, dehydration of leaf cells induced a significant reduction in their content in both types of triticale. Water stress did not significantly affect the content of 3,4‐dihydroxybenzoic acid and gentisic acid in either triticale type (Table 4).

**TABLE 4:** Changes in the content of soluble phenolics (ng/mg [DW]) and cell wall‐bound phenolics (ng/mg [DW]) in triticale with wheat‐dominated genome (WDG) and rye‐dominated genome (RDG).

Phenolic acids	Soluble phenolics		Cell wall‐bound phenolics	
	WDG	RDG	WDG	RDG
Gallic				
C	18.9 ± 0.5a	22.5 ± 2.1a	1.27 ± 0.13a	2.20 ± 0.16b
WS	37.3 ± 0.9b	21.5 ± 3.3a	1.86 ± 0.10b	1.34 ± 0.07a
Benzoic				
C	—	—	23.8 ± 1.2a	18.4 ± 2.1b
WS	—	—	16.5 ± 0.8b	11.3 ± 0.5c
3,4‐dihydroxybenzoic				
C	2.0 ± 0.4a	2.2 ± 0.3a	2.25 ± 0.15a	1.70 ± 0.14b
WS	6.0 ± 0.6b	6.8 ± 0.37b	1.30 ± 0.13c	1.08 ± 0.07c
*p* ‐hydroxybenzoic				
C	0.19 ± 0.01a	0.28 ± 0.02c	2.96 ± 0.15a	3.36 ± 0.08a
WS	0.35 ± 0.01b	0.49 ± 0.02d	3.34 ± 0.09a	4.50 ± 0.30b
Gentisic				
C	7.1 ± 0.1a	5.0 ± 0.6b	1.00 ± 0.05a	0.90 ± 0.07a
WS	7.3 ± 0.4a	3.9 ± 0.3b	1.45 ± 0.14b	1.33 ± 0.12b
Caffeic				
C	16.9 ± 1.1a	8.3 ± 0.4b	2.76 ± 0.13a	2.65 ± 0.06a
WS	16.2 ± 1.2a	5.5 ± 0.3c	2.68 ± 0.13a	2.71 ± 0.13a
Vanillic				
C	1.3 ± 0.1a	1.4 ± 0.1a	11.2 ± 0.9ab	9.4 ± 0.9a
WS	3.0 ± 0.2b	2.2 ± 0.2c	13.0 ± 0.4b	12.4 ± 0.9b
Chlorogenic				
C	70.0 ± 0.5a	22.9 ± 0.6c	2.268 ± 1.047a	0.416 ± 0.182b
WS	43.5 ± 1.0b	14.3 ± 0.8d	0.004 ± 0.004b	0.018 ± 0.018b
Homovanillic				
C	2.7 ± 0.1a	3.4 ± 0.1c	4.63 ± 0.43a	5.20 ± 0.14ab
WS	5.6 ± 0.2b	6.0 ± 0.1d	6.46 ± 0.23b	7.34 ± 0.47b
Syringic				
C	1.16 ± 0.03a	0.37 ± 0.02b	0.61 ± 0.06a	0.96 ± 0.07ab
WS	1.22 ± 0.12a	0.90 ± 0.01c	1.16 ± 0.11b	2.01 ± 0.24c
*p*‐coumaric				
C	21.5 ± 0.5a	15.2 ± 1.5c	484.5 ± 16.2a	363.1 ± 22.4b
WS	26.4 ± 0.9b	16.6 ± 0.9c	370.6 ± 29.1b	438.6 ± 26.4ab
Ferulic				
C	15.0 ± 0.2a	12.0 ± 0.4c	1765 ± 52a	1648 ± 35a
WS	20.3 ± 0.2b	15.3 ± 0.2a	1310 ± 99b	1394 ± 68b
Sinapic				
C	2.1 ± 0.2a	6.0 ± 0.3c	—	—
WS	3.4 ± 0.3b	10.1 ± 0.3d	—	—
Salicylic				
C	2413 ± 86a	2296 ± 55 ac	1.42 ± 0.04a	1.21 ± 0.02b
WS	2082 ± 43b	2112 ± 60bc	1.09 ± 0.10bc	0.99 ± 0.08c
Rosmarinic				
C	15.5 ± 0.3a	9.2 ± 1.1c	92.6 ± 3.6a	92.5 ± 3.6a
WS	17.7 ± 0.6b	17.4 ± 0.5ab	70.5 ± 5.0b	63.7 ± 1.4b
Cinnamic				
C	3.2 ± 0.1a	4.0 ± 0.3b	12.3 ± 0.1a	11.1 ± 0.4ab
WS	4.1 ± 0.1b	3.0 ± 0.1a	10.6 ± 0.5b	11.2 ± 0.6ab

*Note*: Mean values ± SE (*n* = 5). Means indicated with the same letters are not significantly different for individual phenolics (Duncan's multiple range test at *p* = 0.05).

Abbreviations: C, control; WS, water stress.

Specific changes in the content of soluble phenolic compounds induced by water stress (WS) were detected for gallic acid (197% of control in WDG, no changes in RDG), *p*‐coumaric acid (123% of control in WDG, no changes in RDG), caffeic acid (no changes in WDG, 66% of control in RDG), syringic acid (no changes in WDG, 243% of control in RDG), and cinnamic acid (128% of control in WDG, 75% of control in RDG; Table 4). Interestingly, in the RDG type, both for control (C) and water stress (WS), a significantly lower level of soluble gentisic acid, caffeic acid, chlorogenic acid, sinapic acid, *p*‐coumaric acid, and ferulic acid was determined than in the WDG type. Contrary to that, the levels of *p*‐hydroxybenzoic acid, homovanillic acid, and sinapic acid in RDG plants were significantly higher than those in control and stressed WDG plants. Other changes were found for gallic acid and vanillic acid (similar content in C WDG and RDG and significantly lower content in WS RDG than WS WDG), 3,4‐dihydroxybenzoic acid and salicylic acid (similar content in WDG and RDG both control and stress‐exposed plants), rosmarinic acid (significantly lower content in C RDG, similar content in both types under stress), and cinnamic acid (significantly higher content in C RDG and significantly lower content in RDG versus WDG under stress; Table 4).

Quantitative analysis of cell wall‐bound phenolics revealed a significant growth in gentisic acid and syringic acid in both triticale types exposed to water stress (WS; Table 4). At the same time, leaf cell dehydration induced a significant reduction in six phenolic compounds, that is, benzoic acid, 3,4‐dihydroxybenzoic acid, chlorogenic acid, ferulic acid, salicylic acid, and rosmarinic acid in WDG and RDG plants. Water stress did not considerably affect the content of caffeic acid in the WDG or RDG cell walls. Specific changes in the content of cell wall‐bound phenolics under water stress were noted for gallic acid (146% of control in WDG, 61% of control in RDG), *p*‐hydroxybenzoic acid (no changes in WDG, 134% of control in RDG), vanillic acid (no changes in WDG, 132% of control in RDG), homovanillic acid (140% of control in WDG, no changes in RDG), *p*‐coumaric acid (77% of control in WDG, no changes in RDG) and cinnamic acid (86% of control in WDG, no changes in RDG) (Table 4).

RDG plants, both control and stressed, had a lower level of benzoic acid than WDG ones. No significant changes in the content of seven cell wall‐bound phenolics, that is, gentisic acid, caffeic acid, vanillic acid, homovanillic acid, ferulic acid, rosmarinic acid, and cinnamic acid, were found for either control (C) or stress (WS) exposed WDG and RDG plants (Table 4). In control plants, significantly lower control level of wall‐bound phenolics was found in RDG than in WDG, while under WS, both types showed similar content of 3,4‐dihydroxybenzoic acid, chlorogenic acid, *p*‐coumaric acid, and salicylic acid. Other changes were noted for cell wall‐bound *p*‐hydroxybenzoic acid and syringic acid (similar content in C WDG and C RDG, significantly higher content in RDG than WDG under WS) and gallic acid (significantly higher level in C RDG and significantly lower stress‐induced content in RDG as compared with WDG; Table 4).

**FIGURE 4:**
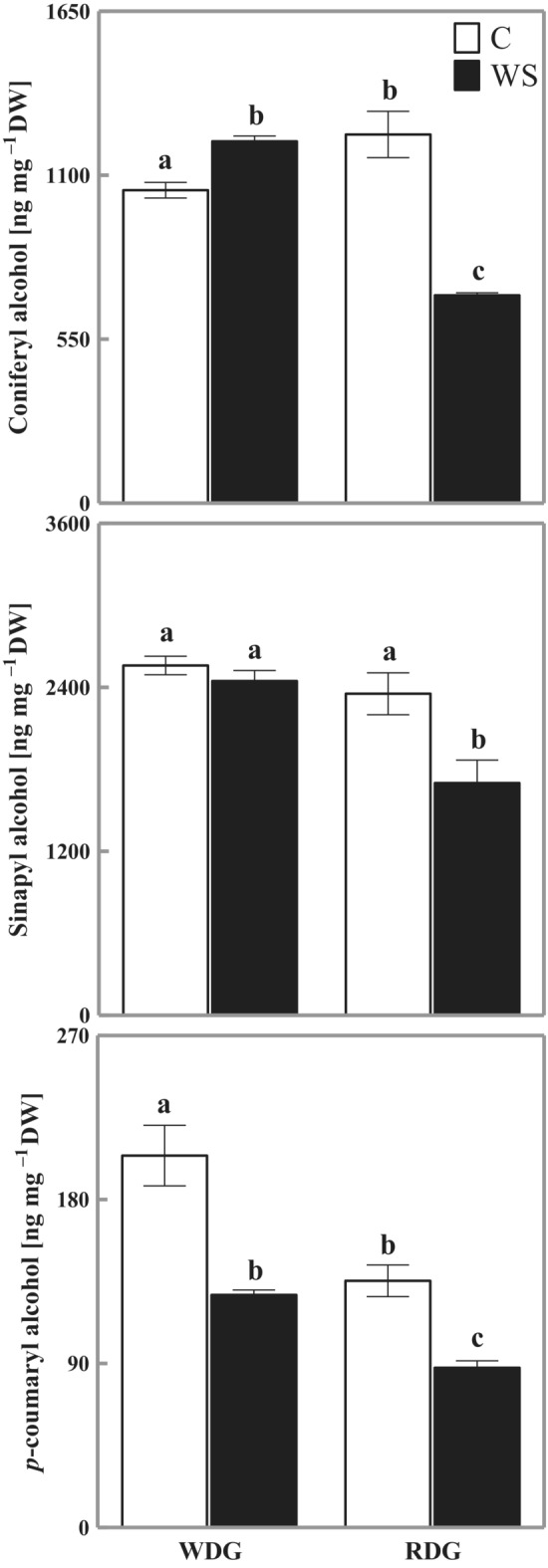
Changes in the content of coniferyl alcohol, sinapyl alcohol, and *p*‐coumaryl alcohol in triticale with wheat‐dominated genome (WDG) and rye‐dominated genome (RDG). C, control; WS, water stress. Mean values ± SE (*n* = 5). Means indicated with the same letters are not significantly different (Duncan's multiple range test at *p* = 0.05).

Quantitative analyzes of monolignols revealed a significant increase in the content of coniferyl alcohol (116% of control) and a significant decrease in *p*‐coumaryl alcohol (63% of control) in the WDG type under water stress (WS; Figure 4). Under the same conditions, no changes in the content of sinapyl alcohol were noted in the WDG type. In RDG plants, water stress induced a significant drop in three analyzed monolignols (56% of control for coniferyl alcohol, 72% of control for sinapyl alcohol, and 65% of control for *p*‐coumaryl alcohol). Under optimal growth conditions (C), WDG and RDG plants significantly differed in their content of coniferyl alcohol (significantly higher content in RDG than in WDG) and *p*‐coumaryl alcohol (significantly lower content in RDG than in WDG), while under water stress, the content of these three monolignols was significantly lower in RDG than in WDG type (Figure 4).

## 4 DISCUSSION

A similar degree of leaf cell dehydration measured based on leaf water potential (Ψ_w_), resulted in greater increase in ABA and considerable limitation of stomatal conductance in WDG plants as compared with RDG ones (Table 1). Accumulation of ABA is a marker of stress intensity (Wang et al., 2022). The level of ABA found in our experiment confirmed the involvement of the rye genome in water stress tolerance (Mubarik et al., 2021). However, it was shown in some species that ABA treatment does not always improve drought tolerance (Bartels & Salamini, 2001; Wu et al., 2020). Other studies also demonstrated that translocation of rye chromosome segment 1RL and 1RS into wheat enhances soil drought tolerance, benefits yielding, and improves water use efficiency by promoting root and above‐ground biomass growth (Ehdaie et al., 2003; Hoffmann, 2008; Karki et al., 2014).

Although water stress did not affect the content of proteins responsible for photosynthetic fixation of CO_2_ and carbohydrate synthesis in WDG and RDG plants, the WDG type accumulated considerably greater amounts of the larger subunit of Rubisco (RbcL; Figure 1A) and SPS (Figure 1B). These results contradict our previous findings on the induction of water stress in the vegetative growth phase of triticale, in which the accumulation of SPS and RbcL was clearly lower in the dehydrated leaves (Hura et al., 2018). The dominance of the wheat genome in the WDG type was evident under optimal growth conditions and it was manifested by significantly higher levels of the analyzed carbohydrates, such as glucose, sucrose, maltose, raffinose, 111‐5‐kestose and nystose, compared to the RDG type (Table 2). The analyses were performed during tillering at the time of intense cell divisions (Yang et al., 2022). Assuero et al. (2012) showed that an increased carbohydrate content in wheat plants might lead to faster leaf and tiller development. Our results for the WDG hybrid indicate that carbohydrate level may be an additional marker for the selection of genotypes with increased tillering potential or productivity associated with high levels of soluble carbohydrates, which may translate into enhanced yielding under optimal growth conditions (Saint Pierre et al., 2010).

Lower carbohydrate content (sucrose, maltose, raffinose, kestose, and 111‐5‐kestose, nystose) in the WDG type under water stress accompanied by accumulation of proteins responsible for carbohydrate synthesis, RbcL, and SPS, may be due to intense consumption of carbohydrates during water stress (Raineri et al., 2015). In the same conditions, the activity of the wheat genome in the WDG type was manifested by a significantly increased content of raffinose and stachyose as compared with the RDG type (Table 2). Raffinose and stachyose are key osmolytes that protect cell components and maintain the osmotic balance in wheat (Kerepesi & Galiba, 2000). Similarly, as in wheat, dos Santos et al. (2011) showed a protective role of raffinose and stachyose accumulation in *Coffea arabica* leaves exposed to osmotic stress. These carbohydrates are also suggested to participate in the scavenging of reactive oxygen species (ROS; Peshev et al., 2013), transport and storage of carbon (Elsayed et al., 2014), mRNA export (Okada & Ye, 2009), membrane trafficking (Thole & Nielsen, 2008), signaling (Stevenson et al., 2000; Xue et al., 2007), as well as stabilizing photosystem II (Knaupp et al., 2011) or other proteins (Bartels & Sunkar, 2005). It should be emphasized that other studies showed accumulation of carbohydrates not only in drought‐tolerant plants but also in drought‐sensitive ones (Nemati et al., 2018; Ozturk et al., 2021).

At the same time, a low DP for fructans and oligofructans (1 < DP <2) indicates that the synthesis of low‐molecular weight fructans and oligofructans dominates in the leaves of both triticale types, irrespective of growth conditions (Table 3). This increases the pool of osmotically active substances that limit cell dehydration during soil drought (Van den Ende, 2013). The analysis of sugar content in both triticale types indicated that water stress could alter not only the level of carbohydrates but also the types of synthesized carbohydrates (Gilbert et al., 1997). Other recent studies based on transcriptomic, proteomic, and metabolomics approaches showed involvement of primary carbon metabolism in plant responses to water stress (Jiang et al., 2021; Kumar et al., 2021).

ShA is an important intermediate in the biosynthesis of secondary metabolites, such as phenolic compounds, involved in plant adaptation to water stress (Hura et al., 2016; Scalabrin et al., 2016). In our study, the decreased level of ShA in WDG plants (Figure 3) indicates a weaker activation of plant defense mechanisms than in RDG type. On the other hand, the increase in ShA content in water stress‐exposed RDG plants could be related to the mobilization of secondary metabolism (Scalabrin et al., 2016). Sicher and Barnaby (2012) also reported about a 10‐fold increase in ShA accumulation under water stress in maize. Other observations were reported for maize (Li et al., 2021) and oil tea (Qu et al., 2019), where plants grown under water stress showed lower levels of ShA.

The metabolism of phenolic compounds was analyzed based on the activity of PAL and TAL. Their activity is affected by environmental stresses (Huang et al., 2010), including water stress (Hura et al., 2009b). A significant increase in the activity of both enzymes in dehydrated leaves was determined only in RDG plants (Figure 2), which indicated a special role of the rye genome in the metabolism of phenolic compounds. This was confirmed in another report (Hura, Tyrka, et al., 2017), which showed that the loci associated with the incorporation of phenolic compounds into the cell wall structures under soil drought are located mainly on rye chromosomes, that is, one locus on Chromosome 3R and two loci on Chromosome 6R. Under the same conditions, in wheat, only 1 locus was located on Chromosome 4B.

The importance of the rye genome in controlling secondary metabolism, analyzed by quantifying phenolic compounds, has been confirmed for several substances. As for soluble phenolic compounds, the RDG type accumulated more *p*‐hydroxybenzoic acid, homovanillic acid, and sinapic acid than the WDG type, both under optimal and limited hydration. Moreover, under optimal conditions, the RDG type accumulated more cinnamic acid (Table 4). These findings may suggest a role of the rye genome in the selective activation of pathways of phenolic compound synthesis that results in the accumulation of specific phenolics (Hura et al., 2016). Enhanced accumulation of *p*‐hydroxybenzoic acid, homovanillic acid, and sinapic acid under water stress was also reported in previous studies (Guo et al., 2020; Quan & Xuan, 2018), including those in triticale (Hura et al., 2016). Recent studies underline the significance of individual phenolic compounds for plant defense mechanisms under water stress conditions (Kravic et al., 2021; Sarker & Oba, 2020).

RDG plants showed higher *p*‐hydroxybenzoic acid and syringic acid content in the cell wall structures than WDG plants (Table 4). Horváth et al., 2002 reported that *p*‐hydroxybenzoic acid increases the impermeability of cell walls, which can limit water loss in plants exposed to water stress (Hura et al., 2016; Hura et al., 2022). It has also been suggested that the increase in the content of phenolic compounds in the cell wall may be due to their inability to form stable bonds with glucose (glucoside synthesis) in the cytosol. This seems to be associated with the low activity of glucosyltransferases specific for individual phenolics (Meyermans et al., 2000). Therefore, the more intense incorporation of these substances into the cell wall structures of the RDG type could result from the limited synthesis of glucosides from *p*‐hydroxybenzoic acid/syringic acid and glucose in the cytosol than in the WDG type.

Monolignols are secreted into the cell wall and cross‐linked through oxidative polymerization. The cross‐linking depends on the availability of ROS generated, among others, by the cell wall peroxidases. This process reinforces the strength and rigidity of cell walls and can be a crucial element of plant response to environmental factors, including water stress (Le Gall et al., 2015). However, our study showed mainly a significant decrease in coniferyl alcohol, sinapyl alcohol, and *p*‐coumaryl alcohol under water stress (Figure 4). This significantly lower level of the three monolignols in RDG and *p*‐coumaryl alcohol in WDG as compared with well‐watered plants could be explained by increased cell wall cross‐linking in this area under water stress (Grabber et al., 2000; Vincent et al., 2005). This way, the cell wall saturated with phenolic compounds improves plant tolerance to water stress (Liu et al., 2018).

In conclusion, water stress in the vegetative growth phase of the intergeneric hybrid induced specific physiological and molecular responses related to the dominance of the wheat genome in the WDG type and the rye genotype in the RDG type. The dominance of the wheat genome is mainly manifested by higher activity of the primary metabolism assessed by the level of carbohydrates, while the dominance of the rye genome is related to, among other things, higher activity of the secondary metabolism of phenolic compounds.

In the WDG type, the higher accumulation of RbcL and SPS, as well as the increase of some carbohydrates, can be considered biochemical and molecular indicators of the wheat genome dominance in nonstressed plants. Under stress, the WDG type showed only increased accumulation of RbcL and SPS and a higher content of raffinose and stachyose than the RDG type. In the RDG type, the indicators of the rye genome dominance in nonstressed plants were the increased content of ShA, some free and cell wall‐bound phenolics, and coniferyl alcohol. In dehydrated RDG plants, the dominance of the rye genome was manifested by a higher than in WDG plants activity of PAL and TAL, as well as a higher level of ShA, free and cell wall‐bound *p*‐hydroxybenzoic acid, free homovanillic acid, free sinapic acid, and cell wall‐bound syringic acid. It should be emphasized that water stress in the early growth phase did not induce as strong specific and differentiating responses of the wheat and rye genomes as it did in the generative growth phase of triticale (Hura et al., 2022). Nonetheless, we suggest that both growth phases of this intergeneric hybrid are suitable for further studies on the precise role of the wheat and rye genomes and the identification of specific features and responses resulting from the interaction between these genomes under water stress.

## AUTHOR CONTRIBUTIONS

*Conceptualization*: Tomasz Hura. *Methodology and investigation*: Tomasz Hura, Katarzyna Hura, Kinga Dziurka, Agnieszka Ostrowska, and Karolina Urban. *Data curation and analysis*: Tomasz Hura and Katarzyna Hura. *Writing* (*original draft preparation*): Tomasz Hura. *Writing* (*review and editing*): Tomasz Hura, Katarzyna Hura, Kinga Dziurka, Agnieszka Ostrowska, and Karolina Urban. *Funding acquisition and project administration*: Tomasz Hura.

## Supporting information


**Table S1.** Multiple reactions monitoring (MRM) transitions for the analyzed monolignols (coumaryl alcohol ‐CmAh, coniferyl alcohol‐CoAh, and sinapyl alcohol‐SnAh) and shikimic acid (ShA): positive ion mode (+ESI), capillary voltage 4 kV, gas temperature 350 °C, gas flow 12 l/min and nebulizer pressure 35 psi. MassHunter software was used to control the LC–MS/MS system and in data analysis. For MRM parameters optimization MassHunter Optimizer was used. D‐BeA ‐ [2H5]benzoic acid.

## Data Availability

The data that support the findings are available from the corresponding author upon reasonable request.
