# Supplementary material for: Cell dehydration of intergeneric hybrid induces subgenome‐related specific responses
Source: Physiol Plant. 2023 Jan 31;175(1):e13855. doi: 10.1111/ppl.13855 (PMC10108068; doi:10.1111/ppl.13855)
Supplement: Supplementary file 1 — Table S1. Multiple reactions monitoring (MRM) transitions for the analyzed monolignols (coumaryl alcohol ‐CmAh, coniferyl alcohol‐CoAh, and sinapyl alcohol‐SnAh) and shikimic acid (ShA): positive ion mode (+ESI), capillary voltage 4 kV, gas temperature 350 °C, gas flow 12 l/min and nebulizer pressure 35 psi. MassHunter software was used to control the LC–MS/MS system and in data analysis. For MRM parameters optimization MassHunter Optimizer was used. D‐BeA ‐ [2H5]benzoic acid. [file PPL-175-0-s001.docx]

**Table S1.** Multiple reactions monitoring (MRM) transitions for the analyzed monolignols (coumaryl alcohol -CmAh, coniferyl alcohol-CoAh, and sinapyl alcohol-SnAh) and shikimic acid (ShA): positive ion mode (+ESI), capillary voltage 4 kV, gas temperature 350 °C, gas flow 12 l/min and nebulizer pressure 35 psi. MassHunter software was used to control the LC–MS/MS system and in data analysis. For MRM parameters optimization MassHunter Optimizer was used. D-BeA - [2H5]benzoic acid.

| Compound |  | Type of ion | Quantifier and qualifier transition  (precursor/product ions) | Fragmentor voltage (V) | Collision energy (V) | Retention Time (min) | Chromatographic conditions |
| --- | --- | --- | --- | --- | --- | --- | --- |
| D-BeA | ISTD | [M+H]^+^ | 128.1/84.2  128.1/81.2 | 61 | 13  29 | 6.32 | AscentisExpres RP-Amide 2.1 mm×150 mm 2.7 μm, 0.5 ml/min at 60°C  A) 3% ACN in H_2_O 0.01% of HCOOH  B) ACN 0.01% of HCOOH  gradient from 3% A at 0 min to 4% in 6 min |
| CmAh |  | [M-H_2_O+H]^+^ | 133.1/105.1  133.1/77.1 | 89 | 17  33 | 3.98 |  |
| CoAh |  | [M-H_2_O+H]^+^ | 163.1/103.1  163.1/77.1 | 71 | 21  41 | 4.83 |  |
| SnAh |  | [M-H_2_O+H]^+^ | 193.1/161.1  193.1/89.1 | 76 | 13  57 | 5.55 |  |
| BeA-D5 | ISTD | [M-H]^-^ | 126/82.1 | 56 | 13 | 1.44 | Poroshell 120 HILIC-Z 2.1×100 mm, 2,7µm, 0.5 ml/min at 40 °C.  A) ACN with 5% 20mM HCOONH_4_  B) 20 mM HCOONH_4_ in H_2_O  Gradient from 95% A at 0 min to 90% in 1.5 min, hold to 3.5 min, then to 20% A at 4.5 min |
| ShA |  | [M-H]^-^ | 173.1/111.1  173.1/93.1 | 107 | 13  13 | 4.42 |  |
